# Transcriptome profiling analysis for two Tibetan wild barley genotypes in responses to low nitrogen

**DOI:** 10.1186/s12870-016-0721-8

**Published:** 2016-01-27

**Authors:** Xiaoyan Quan, Jianbin Zeng, Lingzhen Ye, Guang Chen, Zhigang Han, Jawad Munawar Shah, Guoping Zhang

**Affiliations:** Agronomy Department, Institute of Crop Science, Zhejiang University, Hangzhou, 310058 China

**Keywords:** Barley, Low N tolerance, RNA-Seq, Genotypes, Differentially expressed genes

## Abstract

**Background:**

Nitrogen (N) is the most common limiting factor for crop productivity worldwide. An effective approach to solve N deficiency is to develop low N (LN) tolerant crop cultivars. Tibetan annual wild barley is well-known for its wide genetic diversity and high tolerance to poor soil fertility. Up to date, no study has been done to illustrate the mechanism of LN tolerance underlying the wild barley at transcriptional level.

**Results:**

In this study, we employed Illumina RNA-Sequencing to determine the genotypic difference in transcriptome profile using two Tibetan wild barley genotypes differing in LN tolerance (XZ149, tolerant and XZ56, sensitive). A total of 1469 differentially expressed genes (DEGs) were identified in the two genotypes at 6 h and 48 h after LN treatment. Genetic difference existed in DEGs between XZ149 and XZ56, including transporters, transcription factors (TFs), kinases, antioxidant stress and hormone signaling related genes. Meanwhile, 695 LN tolerance-associated DEGs were mainly mapped to amino acid metabolism, starch and sucrose metabolism and secondary metabolism, and involved in transporter activity, antioxidant activities, and other gene ontology (GO). XZ149 had a higher capability of N absorption and use efficiency under LN stress than XZ56. The higher expression of nitrate transporters and energy-saving assimilation pattern could be attributed to its more N uptake and higher LN tolerance. In addition, auxin (IAA) and ethylene (ETH) response pathways may be also related to the genotypic difference in LN tolerance.

**Conclusion:**

The responses of XZ149 and XZ56 to LN stress differed dramatically at transcriptional level. The identified candidate genes related to LN tolerance may provide new insights into comprehensive understanding of the genotypic difference in N utilization and LN tolerance.

**Electronic supplementary material:**

The online version of this article (doi:10.1186/s12870-016-0721-8) contains supplementary material, which is available to authorized users.

## Background

Nitrogen (N) is an essential mineral nutrient element for plant growth and development [1], which serves as a constituent of many important macro-molecules, including proteins, enzymes, and several plant hormones [2–4]. On the whole, it is a major limiting factor for crop production in the world. Commonly, plants are often exposed to N deficient conditions with the situation becoming more severe due to increasingly-declined soil fertility and widely planted high-yield crop cultivars. Hence, a great amount of N fertilizer is applied to meet N requirement by crops [3], which not only increases the cost for farmers but also brings the environmental problems. In addition it is well documented that only less than half of the applied N fertilizer is used by crops [5], with the surplus contributing to severe environmental pollution. Therefore, it is extremely imperative and important to develop crop cultivars with high LN tolerance or N use efficiency (NUE), as it is a basic and also the most efficient approach for coping with low N availability in the soil and insufficient N fertilizer supply.

Actually, plants have evolved many adaptive responses for coping with LN condition. Moreover, it was found that such N limitation adaptability in crops is closely associated with their yield performance [6, 7]. Thus, genetic improvement of LN tolerance in crops would be of significance for developing sustainable agriculture. On the other hand, it has been well documented that NUE is a genetically controlled trait, differing dramatically among genotypes, such as in *Arabidopsis* [8], as well as in crops including wheat, rice, maize and barley [9–12]. However, narrower genetic diversity in cultivated barley has become a bottleneck for genetic improvement [13]. On the other hand, the Tibetan annual wild barley, growing in the Tibetan Plateau, which has been proved to be one of the centers of cultivated barley [14], contains accessions with high tolerance to abiotic stresses, such as drought, salinity and potassium deficiency [15–17]. Meanwhile, the wild barley shows generally better adaption to poor soil fertility, and we also have identified some wild barley genotypes with high LN tolerance in a previous study [18]. Therefore, it may be assumed that the wild barley contains some unique mechanisms of LN stress tolerance.

RNA-Seq, one of next-generation high-throughput sequencing technologies, has been widely used recently, due to low background noise, high sensitivity and reproducibility, great dynamic range of expression and base-pair resolution for transcription profiling [19]. Using this technique, transcriptomic profiles of many plants have been dissected under any given conditions, including biotic and abiotic stresses, such as heat stress [20] and drought stress [21]. Recently, this method has been also performed on some crops, *viz* rice, sorghum and cucumber [22–24], to investigate the mechanism of LN stress tolerance.

In our previous research, we found a large genetic variation in LN tolerance among the wild barley accessions [18]. However, up to date, no study has been done to analyze the genetic difference of transcriptomic profiles in response to LN stress. It is imperative for us to reveal the underlying mechanism or to explore the relevant genes of LN tolerance in wild barley. In this study, a comprehensive transcriptome analysis was conducted on two wild barley accessions (XZ149, LN-tolerant and XZ56, LN-sensitive), according to a previous study [18]. The main objectives of the current study were to understand the mechanisms of LN tolerance existed in wild barley; and to determine the signaling pathways and regulatory networks of LN tolerance.

## Methods

### Plant materials and treatments

The experiment was carried out in a greenhouse at Zijingang Campus, Zhejiang University, China. Healthy seeds of two wild barley genotypes (XZ149, LN-tolerant and XZ56, LN-sensitive) were disinfected with 2 % H_2_O_2_ for 30 min, rinsed thoroughly with distilled water 5 times, and then soaked for 6 h at room temperature. Then the seeds were moved onto moist filter papers in germination boxes, which were placed in a growth chamber (22/18 °C, day/night) at dark for 3 days, and then incubated for another 7 days with light. At the second leaf stage (10-day old), the similar seedlings were transplanted into black plastic pots (5 L). Endosperm was removed away from the seedlings when they were transplanted, so as to eliminate nutrient supply from seeds. The used full-strength nutrient solution was the same as that reported by Yang et al. [18]. The pH of the solution was adjusted to 5.8 ± 0.1 with NaOH or HCl as required, and was continuously aerated with pumps and renewed every five days. Low N treatment was initiated on the three-leaf-stage seedlings, with 0.2 mM N as LN treatment and 2 mM N as normal level (control).

For biomass and N content determination, the seedlings were harvested and separated into shoots and roots, at 14 d after LN treatment. All the plant samples were heated at 105 °C for 30 min, dried at 80 °C until their weight remained constant, and then dry weight was recorded. N content was determined using Foss Kjeltec 8400.

In order to know the time course of gene *HvNRT2.1* expression under LN stress, the roots of XZ149 were sampled with three biological replicates at 3 h, 6 h, 12 h, 24 h, 48 h, 4 d, and 8 d after LN treatment, frozen in liquid nitrogen immediately, and stored at −80 °C for use in RNA extraction.

### RNA-Seq sampling, RNA extraction and quality control

For RNA-Seq sampling, the seeds of XZ149 and XZ56 were incubated at the same condition as mentioned above. The samples were taken at 6 h and 48 h after exposure to LN stress (0.2 mM) and control (2 mM), respectively. Roots of four seedlings for each treatment were collected and mixed together at each time point. All samples [totally 16, 2 genotypes (XZ149, LN tolerant and XZ56, LN sensitive) × 2 treatments (LN stress and control) × 2 time points (6 h and 48 h) × 2 biological replications] were prepared for further RNA-Seq analysis.

Total RNA was extracted according to the instructions of miRNeasy mini kit (QIAGEN, Germany). RNA degradation and contamination were monitored on 1 % agarose gels. RNA purity was examined using the NanoPhotometer® spectrophotometer (IMPLEN, CA, USA). RNA concentration was determined using the Qubit® RNA Assay Kit in a Qubit® 2.0 Fluorometer (Life Technologies, CA, USA). RNA integrity was analyzed using the RNA 6000 Pico Assay Kit of the Agilent 2100 Bioanalyzer (Agilent Tecnologies, Santa Clara, CA, USA).

### RNA-Seq library construction, sequencing and reads mapping

Sequencing libraries were generated using the Illumina TruSeq™ RNA Sample Preparation Kit (Illumina, San Diego, CA, USA) following the manufacturer`s instructions. Initially, mRNA was purified from the total RNA using poly-T oligo-attached magnetic beads. Then the purified mRNA was fragmented using divalent cations under elevated temperature in Illumina proprietary fragmentation buffer, and reversely transcribed into cDNA using SuperScript II (Invitrogen, Carlsbad, CA, USA). Illumina paired-end sequencing adapters were ligated for preparation of hybridization after making adenylation of the 3’ ends of DNA fragments. In order to preferentially select cDNA fragments of 150 bp, the library fragments were purified using an AMPure XP system (Beckman Coulter, Beverly, USA). With ligated adapters on both ends, DNA fragments were selectively amplified and enriched. Then PCR products were purified again using an AMPure XP system and quantified by Agilent Bioanalyzer 2100 system. Finally, each final cDNA library was applied on one lane of the Illumina paired-end flow cell for the cluster generation processing, and subsequently sequenced on an Illumina NextSeq 500 platform, thus generating 2 × 75 bp pair-ends reads.

The raw reads were generated through the Illumina data processing pipeline (version 1.8). For further analysis, the clean data were obtained by removing low quality bases, empty reads and adaptor sequences at the 3’ end from the raw reads. Meanwhile, the Q20, Q30, GC contents, and sequence duplication level of the clean data were calculated. We downloaded the barley genome sequence and annotation data, and used the ultra high-throughput short read aligner to align RNA-Seq reads to the barley reference genomes on TopHat (http://tophat.cbcb.umd.edu/), and then identified splice junctions between exons by analyzing the mapping results.

### Identification of the DEGs and validation of RNA-Seq by quantitative RT-PCR

For gene expression analysis, FPKM (fragments per kilo-base of exon per million fragments mapped reads) was calculated at expression level [25]. The difference in expression between control and treatment (two biological replicates per time point) was analyzed using the DESeq R package (1.10.1) [26]. A FDR (false discovery rate) was set as 0.05 for the threshold of DEGs [27].

To confirm the validation of the RNA-Seq results, 1 μg total RNA of each sample for RNA-Seq was used for real-time quantitative PCR assays. After eliminating the genomic DNA contamination, first strand cDNA was synthesized with oligo dT primer and Random 6 mers in a 20 μl reaction (Takara, Japan). All quantitative RT-PCR (qRT-PCR) analyses were done in two biological replicates and three technical replicates using a CFX96 system (Bio-Rad, USA). The PCR profiles were as follows: 30 s at 95 °C for pre-denaturation, 40 cycles of 5 s at 95 °C for denaturation and 30 s at 60 °C for annealing, followed by Melt-Curve analysis (60 °C - 95 °C, 0.5 °C increment for 5 s per step) to test the amplicon specificity. For relative quantification, the comparative CT method was used [28]. The amplification of *HvGAPDH* sequence was used as an endogenous reference to normalize all the data. The gene-specific primers were designed using primer-blast (http:/www.ncbi.nlm.nih.gov/tools/primer-blast/), and all the primers were presented in Additional file 1: Table S1.

### Analysis of GO enrichment and Kyoto Encyclopedia of Genes and Genomes (KEGG)

GO annotation and KEGG analysis for the DEGs were performed using the Blast2GO program [29] and the similar steps as reported by Zeng et al. [30]. The GOs distribution associated with DEGs were then obtained from three levels: biological process (BP), molecular function (MF) and cellular component (CC) [30]. The KEGG maps, which contained the EC numbers and enzymatic functions in many metabolic pathways were available in a variety of formats [31].

### Statistical analysis

Significant differences of physiological traits and gene expression among treatments and genotypes were tested using the Duncan’s Multiple Range Test (DMRT) on data processing system (DPS) statistical software, and the difference at *P* < 0.05 and *P* < 0.01 was considered as significant and highly significant, respectively.

## Results

### Effect of N level on growth performances of two wild barley accessions

XZ149 and XZ56 were considered as LN tolerant and sensitive, respectively, according to a previous study [18]. Although LN treatment caused a significant reduction of shoots dry weight for the two accessions, XZ149 was much less affected than XZ56, with XZ149 and XZ56 showing 7.0 % and 28 % reduction, respectively (Table 1). Meanwhile, LN enhanced root growth increase by 35 % and 28 % for XZ149 and XZ56, respectively (Table 1). The less reduction of shoot dry weight and the much increase in root dry weight both contributed to the higher relative total plant dry weight in XZ149. On the other hand, although there was little difference in shoot N concentration between the two accessions under normal N, XZ149 had significantly higher shoot N concentration than XZ56 under LN (Table 1). As a result, N accumulation of XZ149 was 1.58 times larger than that of XZ56 under LN (Table 1). Obviously the current results proved that XZ149 is more LN tolerant than XZ56.

**Table 1:** Growth performances of two wild barley genotypes XZ149 (Low-N-tolerant) and XZ56 (Low-N-sensitive) at 14 d after low N treatment

Trait		XZ149			XZ56		
		CK	LN	Relative	CK	LN	Relative
Dry weight (mg plant^−1^ DW)	Shoot	208.00a	192.83b	0.93	216.00a	134.00c	0.62
	Root	52.17b	70.33a	1.35	56.58b	72.25a	1.28
	Total	260.17a	263.17a	1.01	272.58a	206.25b	0.76
N concentration (%)	Shoot	5.41a	4.67b	0.860	5.34a	4.27c	0.80
N accumulation (mgplant^−1^DW)	Shoot	11.25a	9.01b	0.800	11.53a	5.72c	0.50

CK: Normal N level (2 mM N); LN: Low N level (0.2 mM N); Relative: LN/CK. For each line, different lowercase letters indicate significant differences (*P*, 0.05) among the treatments and genotypes, *n* = 6

### Identification of DEGs

In order to investigate an appropriate time of sampling for RNA-Seq analysis, we observed the response kinetic of the N starvation responsive gene, *HvNRT2.1*. Relative expression of *HvNRT2.1* was calculated using pair-wise comparison between LN and control at 3 h, 6 h, 12 h, 24 h, 48 h, 4 d, 6 d and 8 d after LN treatment, respectively (Additional file 2: Figure S1). The transcript level of *HvNRT2.1* was slightly increased at 3 h and 6 h under LN stress, after then peaked abruptly at 12 h followed by another little peak at 48 h, and thereafter decreased slowly but still remained on a little higher level at 4 d and 8 d (Additional file 2: Figure S1). The results demonstrated that the roots were capable of sensing LN signal and activating relevant signal transduction as early as 3 h after treatment, resulting in differential expression of the relevant genes, which in turn showed highly significant differences at 12 h and 48 h between the N levels. Accordingly, in view of sampling convenience in time arrangement, we then took the samples at 6 h and 48 h for RNA-Seq analysis.

To obtain an overall view of the early LN responsive transcriptome in the two accessions, RNA samples were prepared from the roots of both accessions at 6 h and 48 h after LN treatment. Gene expression profiles of the wild barley roots under both control and LN conditions were analyzed. For each sample, two biological replicates were performed in sequencing. In total, 265597518 clean reads were obtained in the tested samples. For the most samples, 70 % of the sequenced reads could be uniquely mapped (Additional file 3: Table S2).

**Fig. 1:**
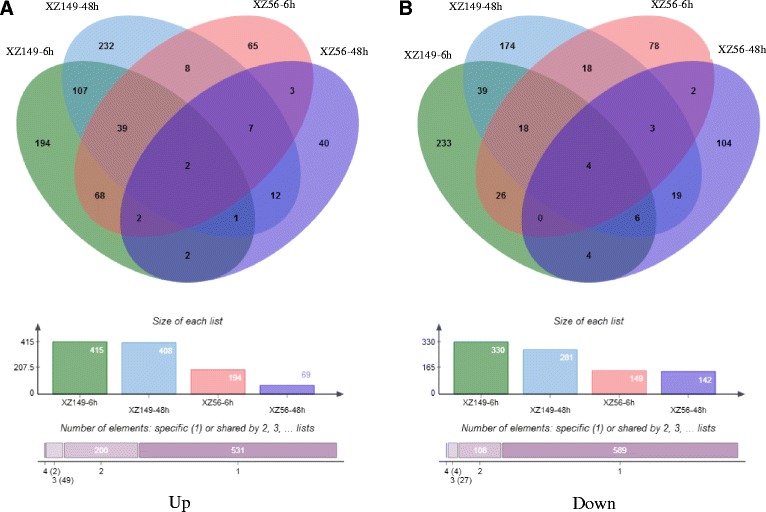
A Venn diagram describing overlaps among differentially expressed genes (DEGs) in XZ149 and XZ56. **a** Up-regulated genes at 6 h and 48 h under low-N treatment. **b** Down-regulated genes at 6 h and 48 h under low-N treatment

The transcriptional levels were normalized using the FPKM method. Meanwhile, FDR < 0.05 was used as screening thresholds to test the significance of difference in transcript abundance. Consequently, 1469 DEGs under LN stress were identified using pair-wise comparison of each accession between normal and low N conditions at each time point (Additional file 4: Table S3). These included both up-regulated (782) and down-regulated (728) genes (Fig. 1). Interestingly, DEGs in the tolerant genotype XZ149 (1203) were nearly twice as much as those in the sensitive genotype XZ56 (524) (Additional file 5: Table S4, Additional file 6: Table S5). However, there were 258 DEGs commonly found in both XZ149 and XZ56. The two accessions displayed dissimilar expression patterns, in which the amount of up-regulated DEGs in XZ56 at 48 h decreased to one third of that at 6 h, while XZ149 maintained little change between 6 h and 48 h (Fig. 1).

**Fig. 2:**
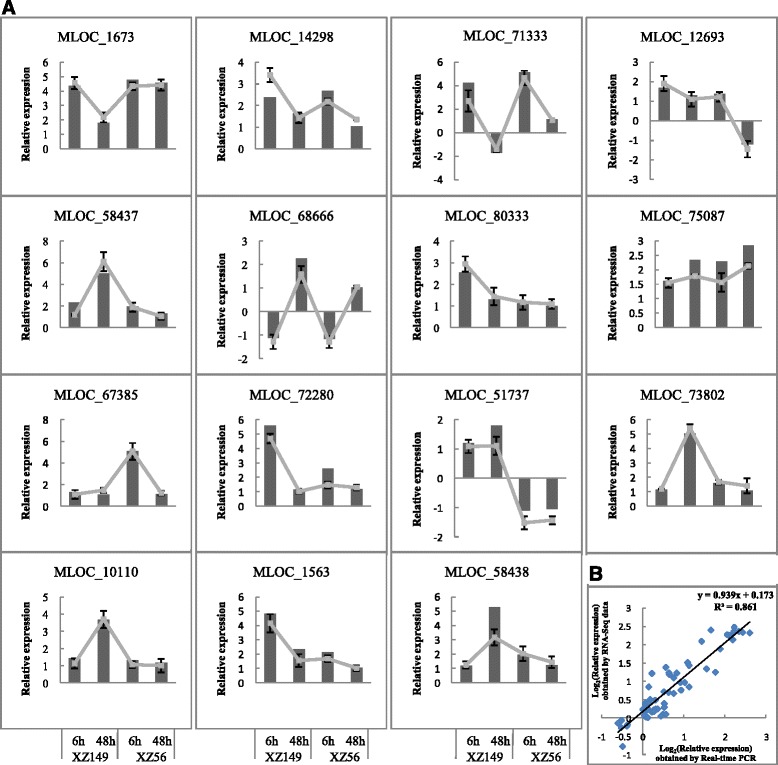
Quantitative real-time PCR validation of 15 differentially expressed genes (DEGs). **a** Transcript levels of 15 DEGs and the corresponding expression data of RNA-Seq. The bars represent SE (*n* = 6). The columns represent relative expression obtained by qRT-PCR, and solid lines represent relative expression obtained by RNA-Seq. **b** Comparison between the relative expression obtained from RNA-Seq data and qRT-PCR. The RNA-Seq log_2_ value of the relative expression (y-axis) has been plotted against the developmental stages (x-axis)

To confirm the validation of the RNA-Seq data, 15 responsive genes were randomly selected for quantitative RT-PCR analysis. The results from both qRT-PCR and RNA-Seq analysis showed that expressions of these genes were highly consistent, thus validating the RNA-Seq data (Fig. 2).

### DEGs involved in nitrogen metabolism

Many genes involved in nitrogen absorption and assimilation were differentially expressed under LN stress relative to the normal condition. In the current study, 12 DEGs encoding nitrate transporters were detected (Table 2). The abundance of these gene transcripts were increased under LN stress. Whereas, the expression patterns of these DEGs in XZ149 differed from those in XZ56, in which transcript levels of most DEGs were increased both in XZ149 and XZ56 at 6 h, and remained being enhanced in XZ149 but declined in XZ56 at 48 h. In addition, three DEGs (MLOC_60308, MLOC_56891, MLOC_51737) were up-regulated only in XZ149, and one DEG (MLOC_65110) was down-regulated only in XZ56. Two LN responsive genes encoding ammonium transporters (MLOC_33834, MLOC_35002) were identified (Table 2), in which, MLOC_35002 was responsive to LN stress only in XZ149. Moreover, seven, one and six DEGs encoding amino acid, lysine histidine and oligopeptide transporters, respectively, were found (Table 2). There were also some DEGs encoding the key enzymes in nitrate assimilation, including two nitrate reductases (NR), one nitrite reductase (NiR) and one glutamate synthase 1 (GOGAT 1) (Table 2). Interestingly, all of these DEGs were down-regulated in XZ149 but remained little changed in XZ56.

**Table 2:** Genes encoding protein transporters and nitrate assimilation enzymes showing genotypic difference expression in response to low N stress. Blank presented in the table means without significant difference in gene expression

Group	Gene id	Log2(Fold change)				Description
		XZ149		XZ56		
		6 h	48 h	6 h	48 h	
Nitrate transporter	MLOC_75087		1.23	1.19	1.51	High affinity nitrate transporter
	MLOC_73802		2.23	0.47	0.72	High-affinity nitrate transporter -like
	MLOC_3053	0.66	1.47	0.70		High-affinity nitrate transporter -like
	MLOC_60308	0.49	0.69			High-affinity nitrate transporter -like
	MLOC_14298	1.25	0.71	1.43		Nitrate transporter
	MLOC_1673	2.14		2.26	2.20	Nitrate transporter
	MLOC_58437	1.22	2.33	0.97		Nitrate transporter
	MLOC_52621	0.95	4.25	1.52	1.10	Nitrate transporter
	MLOC_58438		2.40	0.97		Nitrate transporter
	MLOC_56891	0.61				Peptide nitrate transporter
	MLOC_51737		0.85			Nitrate transporter
	MLOC_65110			−0.80		Nitrate transporter
Ammonium transporter	MLOC_33834	0.61		0.53	−0.56	Ammonium transporter
	MLOC_35002	0.58				Ammonium transporter 3 member 1-like
Amino acid transporter	MLOC_67247		0.57			Uncharacterized amino-acid permease
	MLOC_18832		0.97			Cationic amino acid transporter 5
	MLOC_62449				−0.62	Probable amino acid permease 7 isoform x1
	MLOC_29817	−0.74				Amino acid permease 6-like
	MLOC_36386	−0.69	−1.22	−0.67	−0.69	Amino acid-polyamine transporter
	MLOC_40001		−0.75		−0.98	Sodium-coupled neutral amino acid transporter 2-like
	MLOC_47904				0.57	Amino acid permease 3-like
Lysine histidine transporter	MLOC_54046		0.75			Lysine histidine transporter 1-like
Oligopeptide transporter	MLOC_71333	2.10	−0.78	2.37		Oligopeptide transporter
	MLOC_76910	1.23	−0.68	1.02		Oligopeptide transporter 3-like
	MLOC_16637	0.54	0.76			Oligopeptide transporter 7
	MLOC_16638		1.14			Oligopeptide transporter 7
	MLOC_64771	0.63		0.59		Oligopeptide transporter 7-like
	MLOC_51375	1.04	1.02			Peptide transporter ptr2
Phosphate transporter	MLOC_12153	−1.18		−0.49		Phosphate transporter pho1-2
	MLOC_56639	−0.74				Phosphate transporter pho1-2
	MLOC_6492	−1.02	−1.03			Phosphate transporter pho1-3
	MLOC_28370	1.91		1.24		Phosphate transporter protein
	MLOC_48765	1.38	0.59	1.22		Phosphate transporter protein
Potassium transporter	MLOC_17989	0.75				High-affinity potassium transporter
	MLOC_74879			0.48		Potassium channel akt1-like
	MLOC_13576	−0.67				Potassium channel kor2-like
	MLOC_74565	−0.86	−0.60			Potassium transporter
Sulfate transporter	MLOC_61788	−1.84	−0.91			Probable sulfate transporter
	MLOC_44387		0.52	1.34		Sulphate transporter
Zinc transporter	MLOC_70898	0.86				Zinc transporter 4
Iron transporter	MLOC_57969	1.04	0.84	0.92		Fe(2+) transport protein 1-like
	MLOC_16370	−0.69				Vacuolar iron transporter homolog 5-like
	MLOC_38253	−1.34		−0.56		Vacuolar iron transporter homolog 5-like
	MLOC_36909	−0.52				Vacuolar iron transporter -like
	MLOC_60633	1.39		1.46		Iron-phytosiderophore transporter ysl15-like
	MLOC_5701	0.61	0.48			Metal transporter nramp5-like
	MLOC_61170	0.63		0.91		Metal-nicotianamine transporter ysl3-like
	MLOC_67385			2.36		Metal-nicotianamine transporter ysl6
	MLOC_37227	0.50				Probable metal-nicotianamine transporter ysl12-like
Molybdate transporter	MLOC_61355	0.72				Molybdate transporter 1-like
Nitrate reductase	MLOC_5716	−1.11	−0.70			Nitrate reductase
	MLOC_60358	−0.69				Nitrate reductase
Nitrite reductase	MLOC_43860	−0.80				Ferredoxin--nitrite chloroplastic
Glutamate synthase 1	MLOC_13604	−1.05				Glutamate synthase 1

Meanwhile, expression of many genes associated with absorption or translocation of other nutrients changed under LN stress, such as phosphate (5), potassium (4), sulfate (2), Zinc (1), iron (9) and molybdenum (1), indicating that uptake of these nutrients in barley plants is affected by N metabolism under cross-talking regulation.

### Transcription factors and protein kinases

**Fig. 3:**
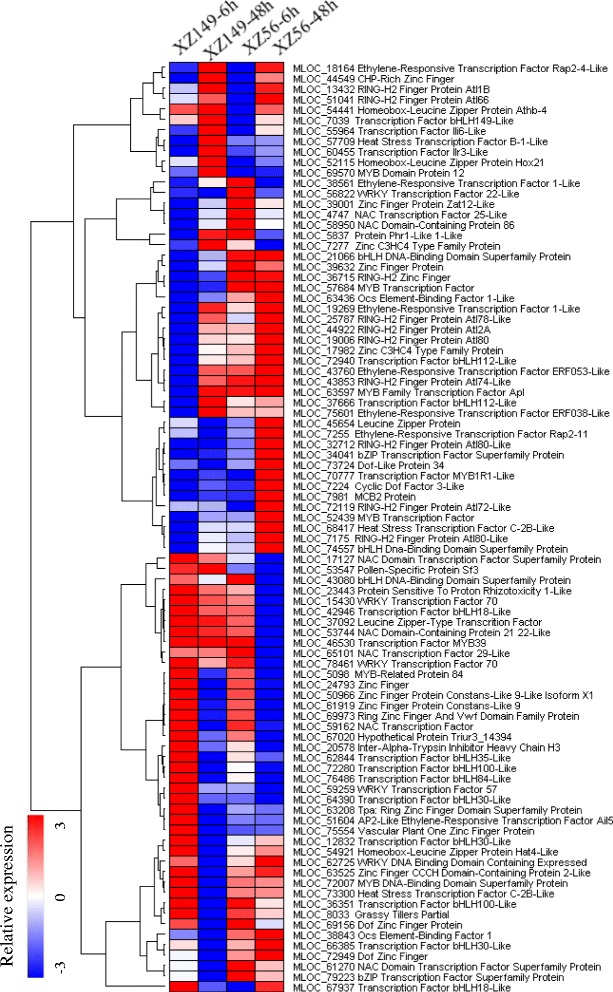
Average linkage hierarchical cluster analysis of transcription factors (TFs) identified in differentially expressed genes (DEGs). The samples and treatments are displayed above each column. Genes are displayed by different colors. Relative levels of expression are showed by a color gradient from low (blue) to high (red)

In total 89 DEGs encoding transcription factors (TFs) were identified in this study, and they belonged to different families, such as Zinc finger (30), bHLH (17), MYB (10), bZIP (10), ERF (7), NAC(7), WRKY (5) and HSF (3) (Fig. 3). XZ149 (75) had more than two fold TFs DEGs than XZ56 (34). Moreover, we found that the proteins with zinc finger domains were the most enriched among the TFs, accounting for 34 % of all these DEGs (Fig. 3).

Kinases play important roles in the development of eukaryotic cells [32], and adaptation to abiotic stresses. Because some of their targets are transcription factors, they also have the functions of regulating transcription [33]. In the current study, 85 DEGs encoding kinases of different groups were identified, which include serine threonine-protein kinase (STK), leucine-rich repeat receptor-like kinase (LRR), lectin protein kinase (LPK), cysteine-rich receptor-like kinase (CRK), CBL-interacting protein kinase (CIPK), wall-associated receptor kinase and calmodulin-binding receptor-like cytoplasmic kinase (CRCK) (Additional file 7: Table S6).

### DEGs related to hormone signaling

In addition to the basic roles in growth and development, phytohormones are also involved in various environmental responses, such as light, salt and drought [34, 35]. It has been proposed that some hormones coordinate demand and acquisition of nitrogen [36]. A total of 47 hormone signaling-related DEGs were found in this study, including gibberellin (GA, 6), cytokinin (CTK, 7), auxin (IAA, 18), ethylene (ETH, 7), abscisic acid (ABA, 8), jasmonic acid (JA, 7), and brassinosteroid (BR, 5) (Additional file 8: Figure S2). Heat map clustering analysis was performed to detect these DEGs involved in hormone signaling. It was found that cytokinin dehydrogenase DEG (MLOC_58639) was up-regulated and remained unchanged in XZ149 and XZ56 under LN stress, respectively (Additional file 8: Figure S2). In addition, three aminocyclopropane-1-carboxylate (ACC) oxidase (ACO) DEGs were up-regulated in XZ149 but remained little changed in XZ56 (Additional file 5: Table S4, Additional file 6: Table S5).

### GO analysis and pathway enrichment analysis of LN tolerance related DEGs

**Fig. 4:**
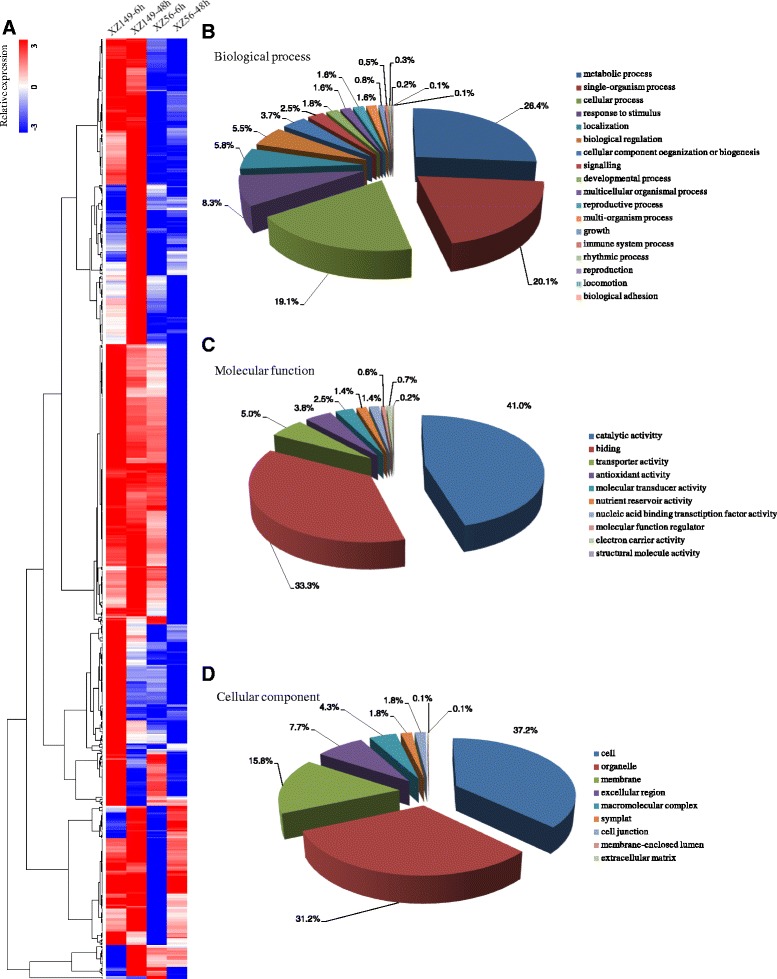
Hierarchical cluster and gene ontology (GO) categories analysis of low N tolerance related DEGs. A total of 695 low N tolerance related DEGs were performed on (**a**) Hierarchical cluster analysis. The samples and treatments are displayed above each column. Genes are displayed by different colors and relative levels of expression are showed by a color gradient from low (blue) to high (red). Gene ontology categories from three levels: (**b**) Molecular function; (**c**) Biological process; (**d**) Cellular component

Of the total 1469 DEGs, 695 DEGs, showing significant up-regulation in XZ149, but down-regulation or unchanged in XZ56, or little change in XZ149 but down-regulation in XZ56, were selected for further investigation. Based on hierarchical clustering analysis, these DEGs could be mainly grouped into four classes. GO functional enrichment analysis were done to classify these DEGs into their corresponding biological process (BP), molecular function (MF) and cellular component (CC) (Fig. 4). The DEGs with known annotation could be categorized into 37 functional groups. In the biological process ontology, GO terms associated with ‘metabolic process’, ‘single-organism process’, and ‘cellular process’, occupied the majority (Fig. 4). The genes associated with catalytic activity and binding were the most enriched, accounting for 74.3 % of molecular function ontology (Fig. 4). Meanwhile, DEGs related to LN tolerance also acted as diverse cellular components (Fig. 4).

**Fig. 5:**
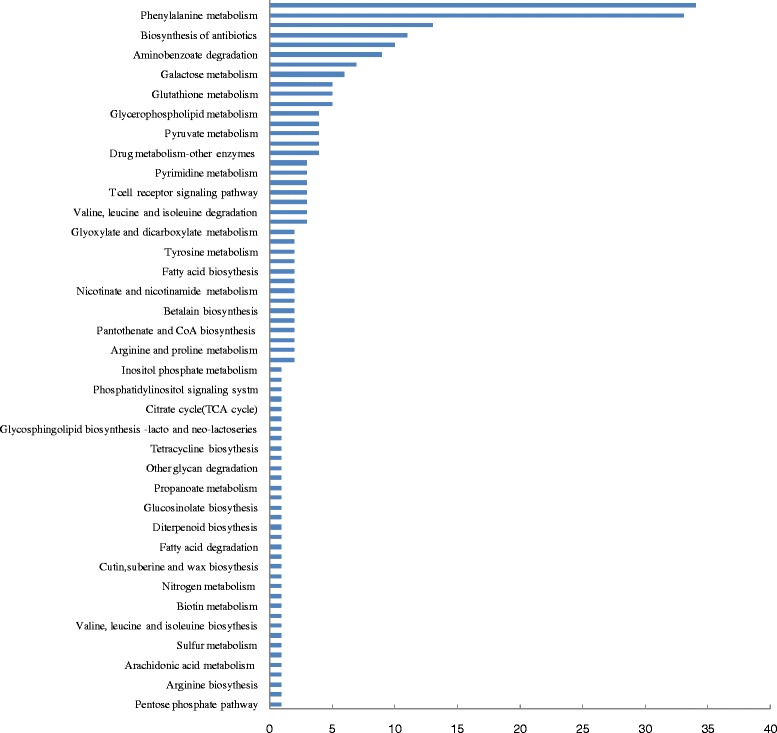
KEGG overview of low-N tolerance related DEGs under low-N stress. X-axis represents the number of DEGs involving in each pathway; Y- axis depicts the different pathway

In addition to GO analysis, 695 DEGs were mapped to terms in KEGG pathway enrichment, and the encoded enzymes were assigned to 72 KEGG pathways (Fig. 5), including amino acid, nucleotide, lipid, carbohydrate, energy and other metabolisms. Among these pathways, DEGs involved in phenylpropanoid biosynthesis (34), phenylalanine metabolism (33), and starch and sucrose metabolism (13) were the most abundant. DEGs (MLOC_4686, MLOC_14829) (Additional file 4: Table S3) encoding the two key enzymes phenylalanine ammonia-lyase (PAL) and cinnamate 4-hydroxylase (C4H), respectively, involved in phenylalanine metabolism, as well as DEG (MLOC_6177) encoding flavonoid 3'5'-hydroxylase (F3'5'H) homolog, were up-regulated only in XZ149 but not changed in XZ56 under LN stress. In addition, the DEG (MLOC_68184) encoding chalcone isomerse (CHI) was down-regulated only in XZ56 and had little change in XZ149. Ten enzymes encoded by 13 LN tolerance-related DEGs were found to be associated with starch and sucrose metabolism.

## Discussion

Nitrogen is an essential mineral nutrient required for plant growth and development. However, N deficiency in soil is becoming a big issue in crop production worldwide. It is imperative to develop the crop cultivars with high LN tolerance or NUE for coping with the issue. There is a large difference among species or genotypes within a species in LN tolerance. Relative biomass or dry weight is often used as an indicator of plant tolerance to low nutrition stress [37, 38]. In the present study, the differences in growth performance between the two wild barley genotypes proved our previous result that XZ149 is more tolerant to LN stress than XZ56, and the results also indicated that XZ149 had the higher capability of N absorption and translocation than XZ56. Meanwhile, we used the RNA-Seq to reveal the differences of the transcriptome profiling between the two wild barley genotypes under low N stress. Clearly, there is a distinct difference in the transcriptional level between the two genotypes in their responses to LN.

### Nitrogen metabolism genes responsive to LN stress

Nitrate transporters are responsible for nitrate absorption from soils. On the whole, N deficiency enhances the expression of high affinity transport systems for nitrate [39]. It was reported that high affinity nitrate transporters *AtNRT2.1*, *AtNRT2.4* and *AtNRT2.5* were induced in N-starved *Arabidopsis* roots [40, 41]. Similarly, the current study showed that the nitrate transporter DEGs were up-regulated under LN stress (Table 2). Moreover, three nitrate transporter DEGs were up-regulated only in XZ149, and in general, the relative increase in abundance of most nitrate transporter DEG transcripts last longer in XZ149 than in XZ56. This unique and higher expression of nitrate transporters in XZ149 may contribute to higher nitrate uptake efficiency, producing more N-containing metabolites required for its survival under LN stress. Thus, it could be assumed that high LN stress tolerance observed in XZ149 is described to its more N uptake and accumulation in plants.

Nitrogen assimilation is another fundamental biological process in plants, which is very energy consuming [42]. The energy cost is particularly larger when nitrate is used as a major N source. When inorganic N is assimilated in roots, energy and C skeletons are provided through respiration, in which sucrose must be supplied from source leaves [43]. Thus, the location of nitrate assimilation may affect energy budget in plants. Against the backdrop, it is reasonable that N assimilation related DEGs (Table 2) in the roots of the tolerant genotype XZ149 were depressed under LN condition, so as to keep energy supply more efficient than transporting sucrose from source leaves to roots for generating ATP and NAD(P)H used in nitrate assimilation. Alternatively, its nitrate and ammonium may be assimilated in the shoots after their transportation out of the roots via the xylem. This less energy-consuming counter measure may also contribute to XZ149’s tolerance to LN stress.

### Carbon metabolism and reducing equivalents

In order to survive under LN stress, some genes related to alleviation of the detrimental effect are abundantly expressed, thus resulting in enhanced stress tolerance. Currently, the transcription of soluble acid invertase (MLOC_60412) was increased in XZ149 but not in XZ56 (Additional file 5: Table S4), indicating that sucrose degradation was enhanced in the tolerant genotype. The similar findings were observed in drought and LN stresses in other studies [23, 44]. Therefore, we may assume that the enhanced invertase expression in the roots of the tolerant genotype XZ149 may stimulate the cycling of sucrose and carbon partitioning in favor of sucrose accumulation for counteracting the LN stress [4].

6-Phosphogluconate dehydrogenase (6PGDH) is a key enzyme involved in the pentose phosphate pathway, where it catalyzes the conversion of 6-phosphogluconate to ribulose-5-phosphate, with the generation of NADPH. In the current study, the DEGs (MLOC_80338) encoding this enzyme were up-regulated in XZ149 (Additional file 5: Table S4), but unchanged in XZ56. As one of the primary end products of the pentose phosphate pathway, NADPH is necessary for fatty acid synthesis, and is needed in response to oxidative stress. Apart from that, it also helps to maintain the reduced state of glutathione (GSH) by serving as co-substrate for glutathione reductases that reduce oxidized glutathione.

### Transcription factors

Several TFs have been described in plants exposed to limited N. For example, Peng *et al*. found that NAC29 showed elevated expression at N deficiency [45]. An R2R3-type MYB TF, CmMYB1, enhances the expression of CmNRT, CmNAR, CmNIR, CmAMT, and CmGS under N-starvation [46]. It was reported that in fungi some GATA factors were involved in regulation of N metabolism and may activate expression of N catabolic enzymes during N-deficiency [47]. However, little has been known about their functions in plants. Here, we found one GATA type zinc finger transcription factor family protein DEG (MLOC_53547) to be up-regulated only in XZ149 (Fig. 3). In addition, three MYB and three NAC transcription factors were found only up-regulated in XZ149 or only down-regulated in XZ56 (Fig. 3). Obviously, it will be quite imperative to determine the possible roles of these TFs in LN stress tolerance in the future.

### Hormone signaling

As a hormone, CTK (cytokinin) acts as a critical signalling molecule in communicating N availability in plants [48, 49]. It has been well documented that CKT is involved in the repression of high-affinity NO_3_^−^ transporter genes and root-shoot-root signaling in *Arabidopsis* [50, 51]. In this study, cytokinin dehydrogenase DEGs (MLOC_58639) involved in CTK degradation pathway was up-regulated and not changed in XZ149 and XZ56 under LN stress, respectively (Additional file 8: Figure S2). Obviously, the regulation of CTK homeostasis in both accessions is consistent with the expression profile of high-affinity NO_3_^−^ transporter genes.

Interestingly, we found that three ACO homologs were increased in XZ149 but remained little changed in XZ56 (Additional file 5: Table S4, Additional file 6: Table S5). It is well known that ACO, a rate-limiting enzyme in ethylene synthesis, can oxidize ACC (the direct precursor of ethylene synthesis) to ethylene,and ACO4 and another ACO homolog had responses to N deficiency [45, 52]. Recent investigations on *Arabidopsis* detected a negative feedback loop between *NRT2.1* expression and ethylene biosynthesis under low nitrate level [53]. Thus we deduced that it may be the finely tuning between up-regulation of *NRT2.1* expression and ethylene biosynthesis in XZ149 should be beneficial for its LN tolerance.

### Antioxidant stress

Recent study visualized reactive oxygen species (ROS) accumulation when Arabidopsis roots were subjected to N deprivation [54], due to the reduction in the frequency of electron carriers of electron transport systems [55]. Excessive accumulation of ROS in plant cells can result in oxidative stress, a major cause of cellular damage and cell death [56]. To cope with this, plants have developed numerous strategies for the detoxification of ROS. Glutathione S-transferases (GSTs) can directly scavenge peroxides with the help of glutathione as electron acceptor [57]. In this study, three DEGs encoding putative GSTs were up-regulated only in XZ149 (Additional file 9: Table S7). In addition, Cytochrome P450s (CYPs), which are involved in biosynthesis and detoxification of a wide variety of molecules [58], had higher expression in rice [59] and sorghum [23] under LN stress, indicating their specific effect of N-induced CYPs on the antioxidant level. Currently, we found nine CYPs DEGs were up-regulated in XZ149, but unchanged in XZ56, and another three unchanged in XZ149, but down-regulated in XZ56 under LN stress (Additional file 9: Table S7). The expression of one gene (MLOC_75745) for aldehyde dehydrogenase (ALDH) was up-regulated only in XZ149 under LN stress (Additional file 9: Table S7). Moreover, a large amount of peroxidases, up to 29, were up-regulated only in XZ149, apparently contributing to the higher capacity of antioxidant defense in XZ149 (Additional file 9: Table S7). Clearly, XZ149 may develop the higher ability of scavenging excessive ROS through forming a stronger antioxidant system under LN stress, facilitating higher tolerance to LN stress.

### Phenylpropanoid metabolism pathway regulated by PAL under LN stress

Phenylalanine is a key amino acid at the interphase of primary and secondary metabolism, and PAL is an initial rate limiting enzyme in phenylpropanoid synthesis [60]. In addition to its important role in plant development, PAL also acts as a key enzyme in response to stress. Its biosynthesis would be stimulated in plants exposed to pathogenic attack, low temperature, salt stress, and low nitrogen, phosphate, or iron [60]. Therefore, PAL has generally been considered as one of the main markers of environmental stress [61]. Possibly, the response to N deficiency could be partly altered through regulation of PAL. In our study, DEG (MLOC_4684) encoding PAL was found only in XZ149 under LN, suggesting that the phenylpropanoid metabolism pathway mediated by PAL regulation may confer to genotypic difference in LN tolerance. Furthermore, lots of DEGs, up to 34, related to LN tolerance, were assigned to phenylpropanoid metabolism pathway according to KEGG metabolic pathway enrichment (Fig. 5). It may be assumed that the enhanced phenylpropanoid metabolism observed in XZ149 under LN stress may contribute to its high tolerance.

### Regulatory network of the flavoniod synthesis pathway under LN stress

LN stressed plants shows significant reduction of photosynthetic capacity and become more susceptible to oxidative damage caused by excessive light [62]. As an adaptive strategy, synthesis of photo-protective pigments, such as anthocyanins and flavonols could be enhanced in plants exposed to LN [63, 64], because anthocyanins may act as the ‘sunscreen’ and scavenger of ROS [62], and flavonols could filter off damaging wavelengths of radiation [65].

**Fig. 6:**
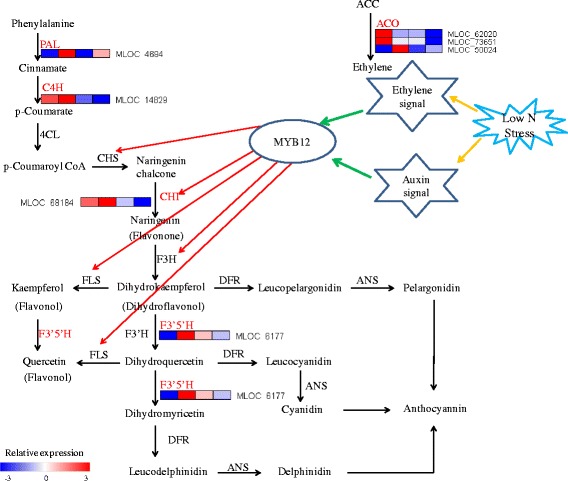
The phenylpropanoid pathway for the biosynthesis of anthocyanin and flavonol. p-Coumaroyl CoA is at the junction of the metabolic routes leading to flavoniods synthesis. Genes are displayed by different colors. Relative levels of expression are showed by a color gradient from low (blue) to high (red). For each heatmap from left to right: XZ149-6 h (first column), XZ149-48 h (second column), XZ56-6 h (third column), XZ56-48 h (fourth column)

Flavonols and anthocyanins synthesis shared the first two phases. Structural genes in anthocyanins and flavonols synthesis specially displayed higher transcriptional level under LN stress in the tolerant genotype XZ149 (Fig. 6). Starting from the first shared phase phenylpropanoid pathway, the DEGs encoding PAL, C4H and F3’5’H were up-regulated only in XZ149 and remained little changed in XZ56 under LN stress (Fig. 6). Moreover, the DEGs (MLOC_68184) encoding CHI were down-regulated only in XZ56 and had little change in XZ149 (Fig. 6).

In addition to the altered structure genes, the regulatory genes in anthocyanins and flavonols synthesis were also affected by LN stress. A *MYB12* orthologous gene (MLOC_69570) was up-regulated in XZ149 under LN stress, while remain unchanged in XZ56. The MYB12 transcription factor, known to be a specific activator of flavonoid metabolism, activated the expression of chalcone synthase (CHS), CHI, flavanone 3-hydroxylase (F3H), and flavonol synthase (FLS) in Arabidopsis [66], and was also intersection of signaling pathways for auxin- and ethylene-mediated flavonol increases [67], indicating that the ethylene and auxin signalling may play an important role in low-N-induced flavonoid synthesis in barley roots. Moreover, three ACO homologs were up-regulated in XZ149 but unchanged in XZ56. Besides, two ethylene-responsive transcription factors (ERFs) (MLOC_18164, MLOC_75601) were unchanged/increased in XZ149 while decreased/unchanged in XZ56 (Fig. 3).

In short, the current results strongly suggest that higher flavonoid accumulation in the tolerant genotype XZ149, especially the accumulation of anthocyanins and flavonols, may be attributed to its higher LN tolerance.

## Conclusion

**Fig. 7:**
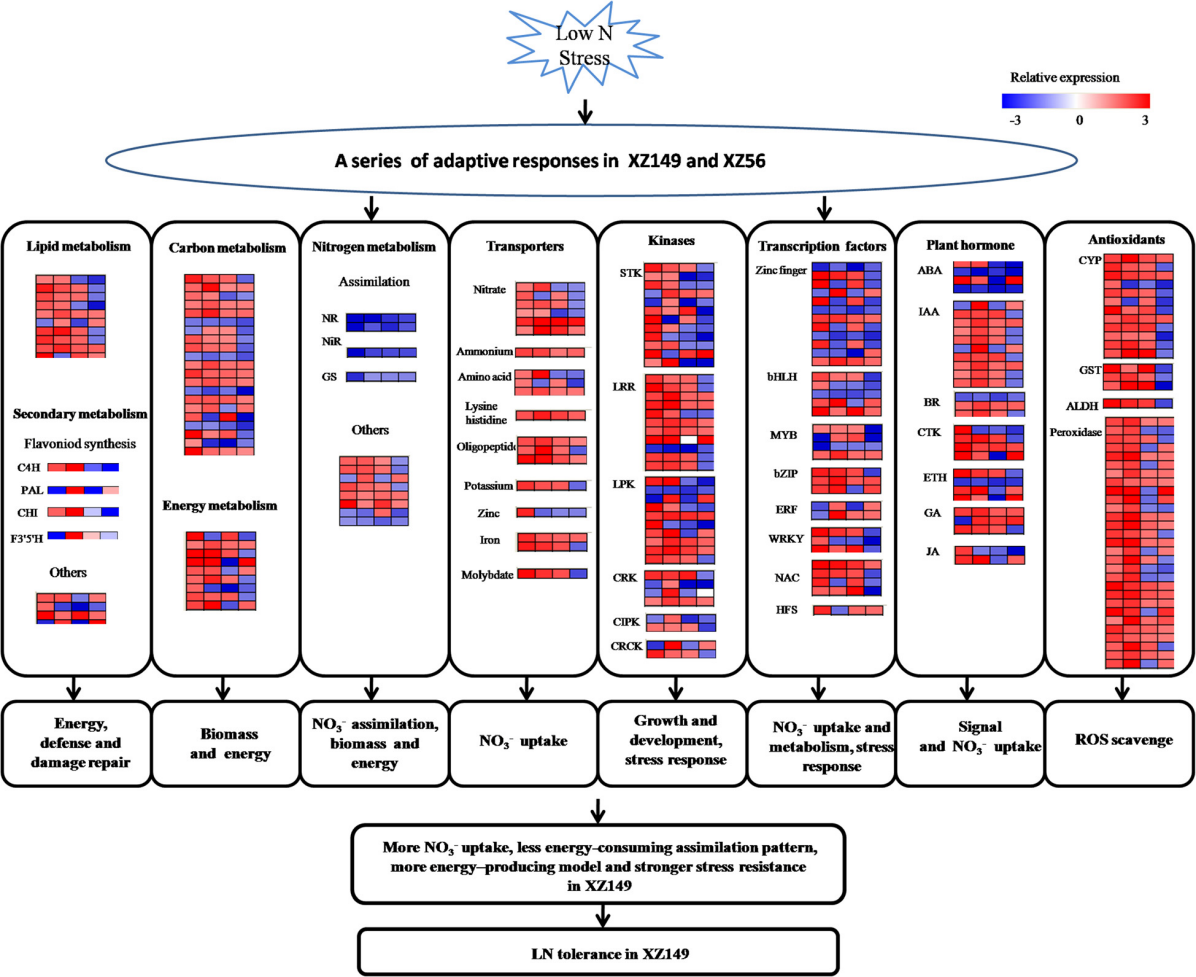
A hypothetical model of low N tolerance mechanism underlying in XZ149. Genes are shown by different colors and relative expression levels are shown by a color gradient from low (blue) to high (red). For each heatmap from left to right: XZ149-6 h (first column), XZ149-48 h (second column), XZ56-6 h (third column), XZ56-48 h (fourth column)

Identification of DEG transcripts in plants under LN stress would reveal the genetic mechanism of LN tolerance. Here, the results of RNA-Seq analysis (all the sequences of the DEGs were available in Additional file 10: Table S8) demonstrated that there was the dramatic difference at transcriptional level between the two Tibetan wild barley genotypes in response to LN stress. Accordingly, a hypothetical model was developed for the mechanism of LN tolerance in XZ149 (Fig. 7). We deduced that more N absorption, less energy-consuming N assimilation pattern, more energy-producing model, which is contributed to high LN tolerance, may explain its well growth performance under LN stress. In addition, the current study identified some candidate genes related to LN tolerance, and cast a light on comprehensive understanding of the genotypic difference in N utilization and LN tolerance.

### Availability of data and materials

The datasets supporting the conclusions of this article are included within the article and its additional files.

## Additional files

Additional file 1: Table S1. The primers used in quantitative real-time PCR. (DOC 51 kb) Additional file 2: Figure S1. Real-time PCR analysis of the *HvHRT2.1* gene in XZ149 under low N stress*. (DOCX 16 kb)*
Additional file 3: Table S2. Summary of mapping reads of the RNA-Seq. (XLSX 16 kb) Additional file 4: Table S3. The FPKM value of 1469 DEGs in XZ149 and XZ56. (XLSX 464 kb) Additional file 5: Table S4. DEGs at 6 h and 48 h after low N treatment in XZ149. (XLSX 160 kb) Additional file 6: Table S5. DEGs at 6 h and 48 h after low N treatment in XZ56. (XLSX 70 kb) Additional file 7: Table S6. DEGs encoding kinases under low N stress in XZ149 and XZ56. (XLSX 14 kb) Additional file 8: Figure S2. Heat Map analysis of DEGs involved in hormone signaling in XZ149 and XZ56. (DOCX 76 kb) Additional file 9: Table S7. DEGs related antioxidant stress under low N stress. (DOCX 16 kb) Additional file 10: Table S8. Gene accession numbers and sequences of 1469 DEGs. (XLS 2369 kb)

## Abbreviations

4CL: p-Coumaroyl CoA synthase
ABA: Abscisic acid
ACC: Aminocyclopropane-1-carboxylate
ALDH: Aldehyde dehydrogenase
ANS: Anthocyanidin synthase
BP: Biological process
BR: Brassinosteroid
C4H: Cinnamic acid 4-hydroxylase
CC: Cellular component
CHI: Chalcone flavanone isomerase
CIPK: CBL-interacting protein kinase
CHS: Chalcone synthase
CRCK: Calmodulin-binding receptor-like cytoplasmic kinase
CRK: Cysteine-rich receptor-like kinase
CTK: Cytokinin
CYPs: Cytochrome P450s
DEGs: Differentially expressed genes
DRF: Dihydroflavonol 4-reductase
ETH: Ethylene
F3H: Flavanone 3-hydroxylase
F3’H: Flavonoid 3-hydroxylase
F3'5'H: Flavonoid 3'5'-hydroxylase
FDR: False discovery rate
FLS: Flavonol synthase
GA: Gibberellin
GO: Gene Ontology
GOGAT 1: Glutamate synthase 1
IAA: Auxin
JA: Jasmonic acid
KEGG: Kyoto Encyclopedia of Genes and Genomes
LN: Low nitrogen
LPK: Lectin protein kinase
LRR: Leucine-rich repeat receptor-like kinase
MF: Molecular function
N: Nitrogen
NR: Nitrate reductase
NiR: Nitrite reductase
PAL: Phenylalanine ammonia-lyase
ROS: Reactive oxygen species
STK: Serine threonine-protein kinase
TFs: Transcription factors
