# Supplementary material for: Transcriptome profiling analysis for two Tibetan wild barley genotypes in responses to low nitrogen
Source: BMC Plant Biol. 2016 Jan 27;16:30. doi: 10.1186/s12870-016-0721-8 (PMC4728812; doi:10.1186/s12870-016-0721-8)
Supplement: Additional file 1: Table S1. — The primers used in quantitative real-time PCR. (DOC 51 kb) [file 12870_2016_721_MOESM1_ESM.doc]

**Table S1. The primers used in quantitative real-time PCR.**

| **Gene ID** | **Description** | **Primer sequence (From 5′ to 3′)** |
| --- | --- | --- |
| MLOC_75087 | High affinity nitrate transporter | F:GGTGCAATGGGGGAATACGA |
|  |  | R:GAGCACACCAAACCCAAACG |
| MLOC_73802 | High-affinity nitrate transporter -like | F:GCGTCACCACCTCCATCAAG |
|  |  | R:ACGGACGAACACCTTGTGA |
| MLOC_1673 | Nitrate transporter | F:GGTCCTCTTCTCCATCTGCG |
|  |  | R:CCCACTCAAGCCCGAAATGA |
| MLOC_51737 | Nitrate transporter | F:TCTTGGCAGATTCCTCGACAA |
|  |  | R:GAGGAAGCACACGGAGGATA |
| MLOC_58437 | Nitrate transporter | F:CTTTGTGGCATGGCGTATCG |
|  |  | R:TTGTCCTTGGCCATGTCTCC |
| MLOC_58438 | Nitrate transporter | F:GTCTCCATATCGCAGGCCAA |
|  |  | R:AGTGCCTTATACGTGCTGGG |
| MLOC_14298 | Nitrate transporter | F:GCTGGCCTCCCTTTCTATCG |
|  |  | R:TGTCATTGTCCCAGCAGAGC |
| MLOC_12693 | Wall-associated receptor kinase 2-like | F:GCTGCGTCACATCATCCCTA |
|  |  | R:TTGAGCTTCCGGGTCACAAA |
| MLOC_68666 | Auxin responsive protein | F:GCTACTCCCTACACTGTGCC |
|  |  | R:GTGCGGTGTGGAGTAATGGA |
| MLOC_80338 | 6-phosphogluconate dehydrogenase | F:GCGTTTCGCCACTTCTACAC |
|  |  | R:TCCAAAACCTTGTCTGGGCA |
| MLOC_10110 | Cytochrome p450 87a3-like | F:CGGAGGGCTATCACATCCAG |
|  |  | R:TTGCGACAAAGTAGGCTGTG |
| MLOC_71333 | Oligopeptide transporter | F:ACAAGAGGAAGAACGGCGAT |
|  |  | R:TGACACTCTACCTCGCACTC |
| MLOC_67385 | Metal-nicotianamine transporter ysl6 | F:AGTCTGAGGACTTTGCAGGTG |
|  |  | R:TTTAGCCGTATGGAAGGGATGG |
| MLOC_72280 | Transcription factor bhlh100-like | F:CTAGGCTGCTACTTCTCGCC |
|  |  | R:ATCCATCACAGGCAGTTGGG |
| MLOC_1563 | Jacalin-like lectin domain containing expressed | F:GCAGACGCAATCCACTCCAT |
|  |  | R:CCGTGTGCTTGTTCCCACTA |
| M36650.1 | GAPDH | F:AAGCATGAAGATACAGGGAGTGTG |
|  |  | R:AAATTTATTCTCGGAAGAGGTTGTACA |
| U34198 | *HvNRT2.1* | F:TCCGCGACAACCTAAACCTC |
|  |  | R:GCATCCATATCGAGGGCCAA |
