# Supplementary material for: Transcriptome profiling analysis for two Tibetan wild barley genotypes in responses to low nitrogen
Source: BMC Plant Biol. 2016 Jan 27;16:30. doi: 10.1186/s12870-016-0721-8 (PMC4728812; doi:10.1186/s12870-016-0721-8)
Supplement: Additional file 2: Figure S1. — Real-time PCR analysis of the HvHRT2.1 gene in XZ149 under low N stress. (DOCX 16 kb) [file 12870_2016_721_MOESM2_ESM.docx]

**Figure S1 Real-time PCR analysis of the *HvHRT2.1* gene in XZ149 under low N treatment*. **** Means significant differences according to the Duncan’s multiple range, P<0.05, n = 4. Primers of *HvNRT2.1* and *GAPDH* for RT-PCR are listed in Table S1.
