# Supplementary material for: Transcriptome profiling analysis for two Tibetan wild barley genotypes in responses to low nitrogen
Source: BMC Plant Biol. 2016 Jan 27;16:30. doi: 10.1186/s12870-016-0721-8 (PMC4728812; doi:10.1186/s12870-016-0721-8)
Supplement: Additional file 8: Figure S2. — Heat Map analysis of DEGs involved in hormone signaling in XZ149 and XZ56. (DOCX 76 kb) [file 12870_2016_721_MOESM8_ESM.docx]

**Figure S2. Heat Map analysis of DEGs involved in hormone signaling in XZ149 and XZ56.** The samples and treatments are displayed above each column. Genes are displayed by different colors. Relative levels of expression are showed by a color gradient from low (green) to high (red).
