# Supplementary material for: Transcriptome profiling analysis for two Tibetan wild barley genotypes in responses to low nitrogen
Source: BMC Plant Biol. 2016 Jan 27;16:30. doi: 10.1186/s12870-016-0721-8 (PMC4728812; doi:10.1186/s12870-016-0721-8)
Supplement: Additional file 9: Table S7. — DEGs related antioxidant stress under low N stress. (DOCX 16 kb) [file 12870_2016_721_MOESM9_ESM.docx]

| **Table S7. DEGs related antioxidant stress under low N stress.** Blank presented in the table means without significant difference in gene expression. | | | | | |
| --- | --- | --- | --- | --- | --- |
| **Group** | **Gene ID** |  | **Log2(Fold change)** | |  |
|  |  | XZ149 | | XZ56 | |
|  |  | 6h | 48h | 6h | 48h |
| Cytochrome P450 | MLOC_10110 |  | 1.88 |  |  |
|  | MLOC_11359 | 0.70 |  |  |  |
|  | MLOC_27605 | 0.80 | 0.57 |  |  |
|  | MLOC_53685 |  |  |  | -0.72 |
|  | MLOC_58793 | 0.54 |  |  |  |
|  | MLOC_61498 |  |  |  | -0.62 |
|  | MLOC_68423 | 1.08 |  |  |  |
|  | MLOC_69260 |  | 0.62 |  |  |
|  | MLOC_69509 | 0.59 | 0.57 |  |  |
|  | MLOC_69571 |  |  |  | -0.78 |
|  | MLOC_71334 | 0.54 | 0.73 |  |  |
|  | MLOC_74781 |  | 1.15 |  |  |
| Peroxidase | MLOC_15687 | 0.54 | 0.76 |  |  |
|  | MLOC_1826 |  | 0.58 |  |  |
|  | MLOC_20243 | 0.59 |  |  |  |
|  | MLOC_213 | 0.56 | 0.66 |  |  |
|  | MLOC_30628 | 0.81 | 1.55 |  |  |
|  | MLOC_31393 |  | 0.59 |  |  |
|  | MLOC_39332 |  | 0.69 |  |  |
|  | MLOC_47615 | 0.61 |  |  |  |
|  | MLOC_48592 |  | 10.00 |  |  |
|  | MLOC_48593 |  | 1.38 |  |  |
|  | MLOC_54892 |  | 0.59 |  |  |
|  | MLOC_55204 | 0.69 | 1.14 |  |  |
|  | MLOC_61102 | 0.72 | 1.16 |  |  |
|  | MLOC_63592 | 0.65 |  |  |  |
|  | MLOC_65210 | 0.89 | 1.10 |  |  |
|  | MLOC_65225 |  | 0.67 |  |  |
|  | MLOC_66375 |  | 1.37 |  |  |
|  | MLOC_66985 |  | 0.70 |  |  |
|  | MLOC_67628 |  | 0.79 |  |  |
|  | MLOC_71338 | 1.13 | 0.73 |  |  |
|  | MLOC_71584 | 0.76 | 0.90 |  |  |
|  | MLOC_74605 | 0.50 | 0.69 |  |  |
|  | MLOC_7633 | 1.49 |  |  |  |
|  | MLOC_7680 |  | 1.10 |  |  |
|  | MLOC_78374 | 0.77 |  |  |  |
|  | MLOC_78725 | 0.61 |  |  |  |
|  | MLOC_79529 |  | 0.98 |  |  |
|  | MLOC_80154 |  | 0.66 |  |  |
|  | MLOC_80183 | 0.53 | 1.32 |  |  |
| Glutathione transferase | MLOC_17677 | 10.00 |  |  |  |
|  | MLOC_19863 |  | 0.54 |  |  |
|  | MLOC_30925 |  | 1.08 |  |  |
| Aldehyde dehydrogenase | MLOC_75745 | 0.65 |  |  |  |
